# Unusual nonlinear switching in branched carbon nanotube nanocomposites

**DOI:** 10.1038/s41598-023-32331-y

**Published:** 2023-03-30

**Authors:** Walter Lacarbonara, Sawan Kumar Guruva, Biagio Carboni, Beate Krause, Andreas Janke, Giovanni Formica, Giulia Lanzara

**Affiliations:** 1 Department of Structural and Geotechnical Engineering, Sapienza University of Rome, Via Eudossiana 18, 00185 Rome, Lazio Italy; 2 Department of Functional Nanocomposites and Blends, Leibniz Institute of Polymer Research Dresden, Hohe Str. 6, 01069 Dresden, Germany; 3 Center for Multi-Scale Characterization, Leibniz Institute of Polymer Research Dresden, Hohe Str. 6, 01069 Dresden, Germany; 4 Department of Architecture, RomaTre University, Via Aldo Manuzio 72, 00153 Rome, Lazio Italy; 5 Department of Engineering, RomaTre University, Via della Vasca Navale 79, 00154 Rome, Lazio Italy

**Keywords:** Carbon nanotubes and fullerenes, Structural properties

## Abstract

In this experimental study, we investigate the nonlinear dynamic response of nanocomposite beams composed of polybutylene terephthalate (PBT) and branched carbon nanotubes (bCNTs). By varying the weight fraction of bCNTs, we obtain frequency response curves for cantilever specimens under harmonic base excitations, measuring the tip displacement via 3D scanning laser vibrometry. Our findings reveal a surprising nonlinear softening trend in the steady-state response of the cantilevers, which gets switched into hardening for higher bCNT weight fractions and increasing oscillation amplitudes. The interaction of bCNTs with the thermoplastic hosting matrix results in stick-slip hysteresis, causing a softening nonlinearity that counteracts the geometric hardening associated with the nonlinear curvature of the first mode of the cantilever. However, when the weight fraction of bCNTs is greater than 1%, the bridging of the branched CNTs leads to the formation of a strong network that contributes to the hardening response at higher oscillation amplitudes. This mechanical behavior is detected by the trend of the nonlinear harmonic spectra and the equivalent damping ratio estimated using the half-power bandwidth method. To predict the observed unusual experimental behavior, we use a nonlinear mathematical model of the nanocomposite cantilever samples derived from a 3D mesoscale hysteretic model of the PBT/bCNT material. Our results suggest that the presence of bCNTs in a thermoplastic matrix is the main driver of the highly tunable nonlinear stiffness and damping capacity of the material. The reported experimental and modeling results provide valuable insights into the nonlinear dynamic behavior of PBT/bCNT nanocomposites and have potential applications in the design of advanced materials with tailored mechanical properties.

## Introduction

Traditional materials are known to become nonlinear beyond the elastic limit which usually occurs at moderately large strains^1^ due to nonlinear hyperelasticity, elasto-plasticity or hysteresis. On the contrary, nanostructured materials made of 0D, 1D, or 2D nanofibers dispersed in a hosting matrix can exhibit a nonlinear response at small strains due to the nanofiber-matrix interfacial interactions. When the 1D nanofibers are carbon nanotubes (CNT), the elastic mismatch between the CNTs and the polymer matrix gives rise to high interfacial shear stresses which can overcome the weak van der Waals CNT-polymer interaction forces and cause interfacial sliding and, with it, energy dissipation^2–4^. This is an important source of material nonlinearity which has been referred to as stick-slip^5–11^. Experimentally acquired nonlinear oscillations in the lowest bending mode of cantilevers made of pure polybutylene terephthalate polymer (PBT) showed^10^ that the response is hardening, as expected, due to the geometric nonlinearity of the bending curvature. On the other hand, oscillations of nancomposite cantilevers made of PBT and single-walled-CNTs were proven to be softening. These results were explained by the softening hysteresis caused by the sliding between the CNTs and polymer chains wrapped around them.

The mechanical effects of branched CNTs dispersed in polymer matrices are less known than those of straight (non-branched) CNTs. In the literature, it is well established that the incorporation of straight CNTs into thermoplastic polymers results in changes of the linear mechanical properties, such as storage and loss moduli^12^. Branched CNTs with T, Y, L, and more complex junctions^13^ are expected to enhance the ability to form a network, since the CNTs junctions are already present in the filler material. The interaction between branched CNTs and the polymer matrix can be greatly enhanced by the presence of side branches in bCNTs due to the higher specific surface area in contact with the polymer chains. These side branches can strongly disrupt the mobility of the polymer chains near the reinforcements, leading to improved mechanical properties^14^.

Liu et al. simulated the addition of patterned CNTs to polyethylene-based composites, resulting in ultra-strong nanocomposites thanks to the dramatic improvement in the interfacial strength between the reinforcement and the matrix^14^. The interfacial strength can be significantly affected by both the molecular weight of the polymer as well as by the geometry of the branched CNTs (number of branch points, length of branches, angle between branches). Simulations and pull-out studies have shown that branched fibers can indeed increase interfacial adhesion^15–18^. Starting from the first synthesis of bCNTs achieved in 1999^19^ by pyrolysis of acetylene in Y-shaped templates, over the years branched CNTs revealed even more surprising material properties. Bonab et al. reported a comparative study of thermoplastic polyurethane (TPU) containing linear CNTs and bCNTs using in-situ polymerization. The composite containing bCNTs was shown to form stronger networks than the linear CNTs composite, leading to better mechanical properties. The enhanced multifunctional properties of nanostructured polymers have opened up a wide range of engineering applications, including the production of lightweight materials for the automotive and aerospace industries, as well as materials for use in thermal and electrical conductors, energy storage devices, sensors, and more (see, e.g.^20–22^). However, while the mechanical and thermal/electrical conductivity properties of bCNT nanocomposites have been widely studied, their dissipative properties and nonlinear mechanical response features have not been as thoroughly explored in the literature. This gap in knowledge highlights the need for further research in these areas, as a better understanding of the nonlinear mechanical behavior and dissipative properties of these materials could lead to the development of even more advanced and innovative engineering applications.

We aim to understand how the nano-scale stick-slip phenomenon, widely investigated in linear CNT nanocomposites^2–4,23–25^, affects the overall nonlinear mechanical response of bCNT/PBT nanocomposites. Moreover, the nonlinear material features of a nanocomposite can be manifested in different ways depending on the activated nonlinearities (e.g., geometric nonlinearities, boundary nonlinearities, etc.). In a wider perspective, it is known that the mechanical response and damping capacity of a nonlinear material system depend not only on the constituent material but also on the structural features of the investigated system. In the literature there are numerous examples of mechanical devices that have been designed to exhibit the desired nonlinear response. One of such examples is a special nonlinear resonator in which the rheological component was designed to intentionally exhibit a softening or hardening response by promoting or demphasizing the phase transitions in the material crystalline microstructure (NiTiNOL) and the macroscale frictional dissipation and geometric nonlinearities^1,26–28^.

In this work, samples made of PBT and bCNTs with various levels of wt% are prepared and characterized. Forward and backward frequency sweeps of PBT/bCNT cantilevers around the resonance of the first bending mode are performed to analyze the nonlinear mechanical response. The experimental results, in agreement with the predictions of a nonlinear mechanical model, show an unusual switching of the response from softening to hardening for relatively high bCNT wt% and high oscillation amplitudes. We believe that these results can be physically explained and pave the way towards new classes of innovative materials which can change the way we design materials and conceive their applications.

## Results

**Figure 1:**
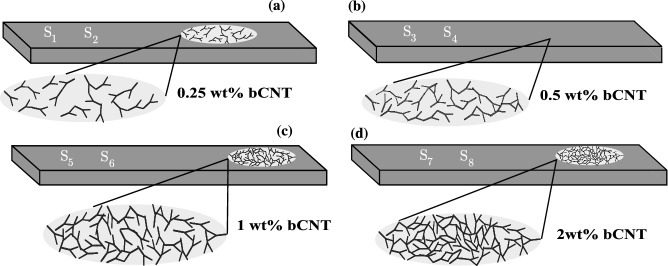
Nanocomposite specimens with different bCNT weight fractions: (**a**) $0.25\%$, (**b**) $0.5\%$, (**c**) $1\%$, (**d**) $2\%$. Two samples of each type were tested. Samples $\text{ S}_{1}$ and $\text{ S}_{2}$ contain 0.25 wt% bCNT, samples $\text{ S}_{3}$ and $\text{ S}_{4}$ contain 0.5 wt%, $\text{ S}_{5}$ and $\text{ S}_{6}$ contain 1 wt%, and samples $\text{ S}_{7}$ and $\text{ S}_{8}$ contain 2 wt%.

**Figure 2:**
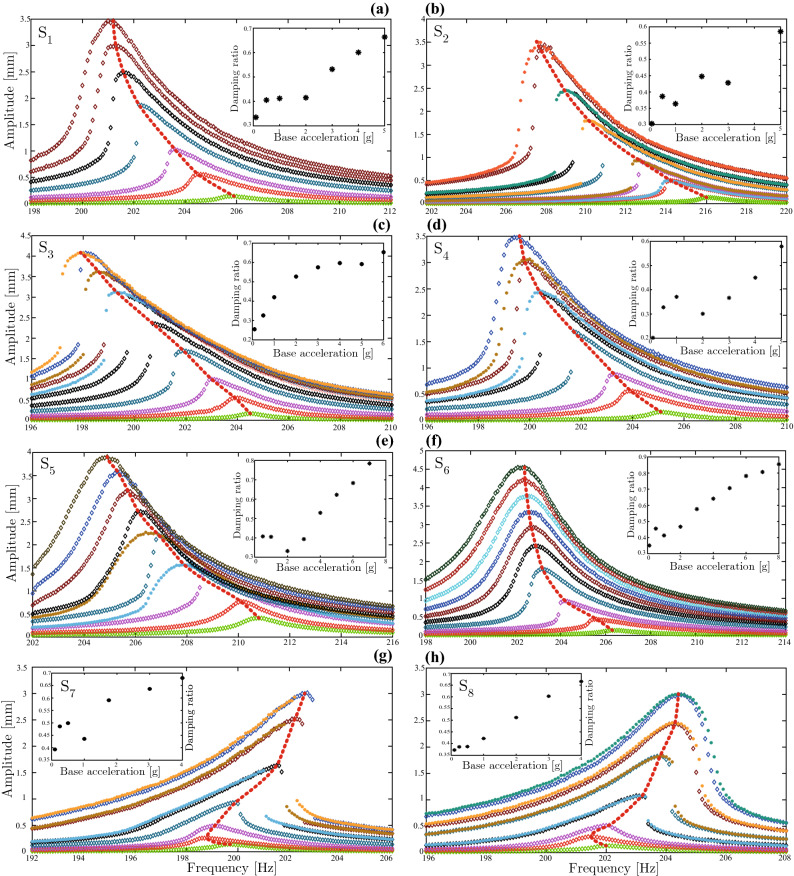
Frequency–response curves and equivalent damping ratios of the lowest bending mode of the cantilever nanocomposite specimens $\text{ S}_1$ through $\text{ S}_8$ with different bCNT weight fractions (0.25 wt%, 0.5 wt%, 1 wt%, 2 wt%) obtained performing forward (diamonds) and backward (asterisks) frequency sweeps for increasing base accelerations: (**a**) (0.1, 0.5, 1, 2, 3, 4, 5)*g* for $\text{ S}_1$; (**b**) (0.1, 0.5, 1, 2, 3, 5)*g* for $\text{ S}_2$; (**c**) (0.1, 0.5, 1, 3, 4, 5, 6)*g* for $\text{ S}_3$; (**d**) (0.1, 0.5, 1, 3, 4, 5)*g* for $\text{ S}_4$; (**e**) (0.5, 1, 2, 3, 4, 5, 6, 7)*g* for $\text{ S}_5$; (**f**) (0.1, 0.5, 1, 2, 3, 4, 5, 6, 7, 8)*g* for $\text{ S}_6$; (**g**) (0.1, 0.25, 0.5, 1, 1.75, 3, 4)*g* for $\text{ S}_7$; (**h**) (0.1, 0.25, 0.5, 1, 2, 3, 4)*g* for $\text{ S}_8$.

PBT/bCNT samples with various bCNT weight fractions were prepared and characterized. Samples named $\text{ S}_{1}$ and $\text{ S}_{2}$ contain 0.25 wt% bCNT, samples $\text{ S}_{3}$ and $\text{ S}_{4}$ contain 0.5 wt%, $\text{ S}_{5}$ and $\text{ S}_{6}$ contain 1 wt%, and samples $\text{ S}_{7}$ and $\text{ S}_{8}$ contain 2 wt% (see Fig. 1). The PBT/bCNT samples were subject to dynamic testing to acquire families of frequency response curves (FRCs) which are shown in Fig. 2 for various excitation magnitudes, together with the loci of the resonance peaks indicated by the red dotted lines. These curves are the best estimate of the so-called backbone curves describing the dependence of the nonlinear frequency on the oscillation amplitude. To ensure a significant nonlinear response, the range of excitation amplitudes was carefully selected based on preliminary computations. This process involved evaluating various amplitudes to determine which ones would result in the desired response. Ultimately, the selected range of excitation amplitudes allowed for the detection and characterization of the nonlinear behavior of the system under investigation.

The displacement peaks *a*, resonance frequencies $f_n$ and equivalent damping ratios $\zeta$ at various excitation levels are reported in Tables 1 and 2 for the two samples $\text{ S}_{1}$ and $\text{ S}_{2}$. The FRCs of $\text{ S}_{2}$ are similar to those of $\text{ S}_{1}$, except for the values of resonance frequencies shown in Fig. 2a. In fact, the highest resonance frequency of $\text{ S}_{2}$ is 216 Hz for the base excitation of 0.1*g* where *g* indicates the gravity acceleration while that of $S_1$ is 205.9 Hz. This suggests that the equivalent elastic modulus of $\text{ S}_{2}$ is slightly larger than that of $\text{ S}_{1}$. The periodic response of both specimens shows jumps due to the fold bifurcations. For $\text{ S}_{1}$, jumps from small amplitude nonresonant responses to large amplitude resonant responses are observed. These FRC curves were obtained performing forward sweeps (see FRCs for 1*g* up to 5*g*). For $\text{ S}_{2}$, also backward sweeps were acquired and a perfect agreement between the forward and backward sweeps was found for the excitation levels from 0.5*g* to 3*g* for which the full set of stable harmonic responses is obtained.

Both $\text{ S}_{1}$ and $\text{ S}_{2}$ exhibit a clear softening behavior for increasing oscillation amplitudes (see Fig. 2a,b). The softening hysteresis is attributed to the CNT/polymer stick-slip and overcomes the hardening effect due to the nonlinear bending curvature which is typically shown by the first bending mode of linearly elastic isotropic polymeric cantilevers^27^. Moreover, the jumps in the response disappear at higher excitation amplitudes indicating that the interfacial sliding between the bCNTs and polymer chains reaches a plateau at large oscillations. The switching of the response from being multi-stable due to the existence of fold bifurcations (multi-valued FRCs) to mono-stable with single-valued FRCs for increasing oscillation amplitudes, can be explained by the growth of the dissipated energy that balances the stored energy. The insets in Fig. 2a,b show the equivalent damping ratios as function of the excitation level for the $\text{ S}_{1}$ and $\text{ S}_{2}$ samples, respectively. The trends of the damping ratio confirm the increase of dissipated energy according to the topological features of the FRCs. The damping ratio of $\text{ S}_{1}$ shows two different trends: from 0.1*g* to 0.5*g* the ratio increases reaching an almost constant value; from 0.5*g* to 5*g* the rate of increase is markedly larger indicating the activation of the stick-slip mechanism (see Fig. 2a,b). The behavior of $\text{ S}_{2}$ is similar to that of $\text{ S}_{1}$ but the threshold excitation level is 3*g*.

The behavior of the $\text{ S}_{1}$ beam sample exhibits a softening trend for small and large oscillation amplitudes as the base excitation level increases from 0.1*g* to 5*g* (see Fig. 2a). In fact, the highest resonance frequency of 205.9 Hz is exhibited for the lowest base excitation of 0.1*g*. For the larger excitation amplitudes, the resonance occurs at increasingly smaller frequencies revealing the softening trend.

**Table 1:** Peak amplitudes, associated resonance frequencies and equivalent damping ratios exhibited by sample $\text{ S}_{1}$ (0.25 wt% bCNT) for different excitation levels.

Excitation (*g*)	$\ f_{n}$ (Hz)$^{\rm a}$	*a* (mm) $^{\rm b}$	$\zeta$ ($\%$) $^{\rm c}$
0.1	205.9	0.1439	0.3349
0.5	204.5	0.5578	0.4063
1	203.6	1.03	0.4134
2	202.2	1.876	0.4167
3	201.6	2.49	0.5333
4	201.3	3.004	0.6020
5	201.0	3.467	0.6641

$$^{\rm a}$$Resonance frequency.

$$^{\rm b}$$ Peak amplitude.

$$^{\rm c}$$Damping ratio.

**Table 2:** Peak amplitudes, associated resonance frequencies and equivalent damping ratios of sample $\text{ S}_{2}$ (0.25 wt% bCNT) for different excitation levels.

Excitation (*g*)	$\ f_{n}$ (Hz)$^{\rm a}$	*a* (mm)$^{\rm b}$	$\zeta$ ($\%$) $^{\rm c}$
0.1	216	0.1353	0.3033
0.5	214.2	0.5034	0.3877
0.5 − b	214.2	0.5034	
1	212.6	0.9323	0.3652
1 − b	212.6	0.9323	
2	210.9	1.616	0.4499
2 − b	210.1	1.806	
3	209.4	2.374	0.4300
3 − b	208.9	2.463	
5	207.9	3.436	0.5877
5 − b	207.5	3.514	

$$^{\rm a}$$ Resonance frequency.

$$^{\rm b}$$ Peak amplitude.

$$^{\rm c}$$ Damping ratio.

The experimental FRCs of samples $\text{ S}_{3}$ and $\text{ S}_{4}$ are shown in Fig. 2c,d, respectively. Also for these two samples, the nonlinear dynamic behavior is characterized by material softening signaled by the fact that the resonance frequency decreases as the excitation level increases. The lowest resonance frequencies of $\text{ S}_{3}$ and $\text{ S}_{4}$ are close denoting a similar distribution of bCNTs in the two samples. Moreover, the resonance frequencies for the lowest excitation (i.e., the linear frequencies) are very close to those exhibited by $\text{ S}_{1}$, thus indicating that increasing the wt% bCNT by 0.5% does not have a remarkable effect on the equivalent elastic modulus. However, this result cannot be generalized since it is mostly related to the achieved overall dispersion of bCNTs for this specific case. This interpretation is confirmed by the fact that the resonance frequency of $\text{ S}_{2}$ obtained for 0.1*g* is larger than that of the other samples.

The comparisons can be performed because the oscillation amplitudes exhibited by $\text{ S}_{1}$, $\text{ S}_{2}$, $\text{ S}_{3}$ and $\text{ S}_{4}$ for the base excitation of 1*g* are practically the same. The nonlinear frequency trends of $\text{ S}_{3}$ and $\text{ S}_{4}$ are similar to those of $\text{ S}_{1}$ and $\text{ S}_{2}$. In particular, $\text{ S}_{4}$ shows the same peculiar behavior of $\text{ S}_{1}$ and $\text{ S}_{2}$ (i.e., the transition from single- to multi- and again single-valued FRCs) that denotes the growth of dissipated energy towards large oscillation amplitudes. The equivalent damping ratio versus base acceleration for $\text{ S}_{3}$ and $\text{ S}_{4}$ is shown in the insets of Fig. 2c,d, respectively. The two different trends, already observed for $\text{ S}_{1}$ and $\text{ S}_{2}$, can be clearly discerned. In particular, the threshold excitation values for which the stick-slip mechanism takes place are 5*g* for $\text{ S}_{3}$ and 2*g* for $\text{ S}_{4}$. The experimental FRCs of samples $\text{ S}_{5}$ and $\text{ S}_{6}$ are shown in Fig. 2e,f, respectively. The periodic responses of $\text{ S}_{5}$ in Fig. 2e exhibit a softening behavior for small and large oscillation amplitudes confirming the trend of the samples with lower bCNT wt%. Furthermore, the shifts of the resonance frequencies towards higher values (especially for $\text{ S}_{5}$) with respect to $\text{ S}_{3}$ and $\text{ S}_{4}$ indicates an increase of the nanocomposite elastic modulus provided by the larger bCNT wt%.

The softening response is pronounced for both $\text{ S}_{5}$ and $\text{ S}_{6}$ considering the entity of the frequency shift from the lowest to the largest excitation level. At the same time, small jumps of the response can be observed only for $\text{ S}_{6}$ at 1*g*. These two aspects denote a high rate of dissipated energy due to stick-slip that increases with the oscillation amplitude. The overlapping of the responses over 1*g* and the difference between the backward and forward sweeps at 2*g* and 3*g* (see $\text{ S}_{5}$ in Fig. 2e) can be mainly explained by the rearrangement of the bCNTs in the PBT matrix which modifies the equivalent tangent elastic modulus of the material. This effect is not exhibited by $\text{ S}_{6}$ that preserves the equivalent elastic properties for all tests. The trends of the damping ratio as function of the excitation level is provided in Fig. 2e,f. For $\text{ S}_{5}$, the transition from the elastic regime (characterized by the absence of sliding between bCNTs and hosting matrix) to the stick-slip regime occurs at 3*g*. On the contrary, this change of behavior cannot be observed for $\text{ S}_{6}$ where, except for the damping value obtained at 0.25*g*, the increasing trend exhibits a constant rate.

The experimental FRCs of samples $\text{ S}_{7}$ and $\text{ S}_{8}$ are shown in Fig. 2g,h, respectively. The steady-state periodic responses of $\text{ S}_{7}$ show a softening trend for small oscillation amplitudes. However, past a threshold excitation amplitude, the beam exhibits a hardening behavior as shown in Fig. 2g. The same behavior can be observed for $\text{ S}_{8}$ and the transition from softening to hardening occurs in both specimens between the excitation amplitudes of 0.25*g* and 0.5*g*. This unusual behavior was predicted by a model of CNT/polymer nanocomposite beam models^29^. The softening characteristic behavior in the small oscillation range, due to the interfacial stick-slip, is contrasted by the geometric hardening associated with the nonlinear bending curvature of the first mode which becomes dominant at large oscillation amplitudes. The decrease of the equivalent damping is also proven by the jumps in the FRCs shown by $\text{ S}_{7}$ at large excitations (i.e., the transition from single- to multi-valued FRCs for increasing levels of excitation). The backward and forward sweeps are in agreement according to the oscillation amplitudes. Sample $\text{ S}_{8}$ shows a peculiar behavior characterized by the transition from single-to multi-valued and again-single-valued responses together with the hardening characteristic. This result suggests that also the interaction between the bCNTs and PBT polymer chains has a role in the stiffening of the material. At the same time, both $\text{ S}_{7}$ and $\text{ S}_{8}$ samples, which contain the largest bCNTs weight fraction, do not exhibit a shift of the resonance frequency at 1*g* with respect to the other samples. This indicates that the increase up to 2 wt% bCNT does not have an appreciable effect on the linear elastic properties. The increase of the equivalent damping exhibited by $\text{ S}_{7}$ (see Fig. 2g) shows a larger rate for the lowest two excitation levels. For 0.25*g*, the lower rate of increase suggests that the bCNTs have a modest mobility. For $\text{ S}_{8}$ (see Fig. 2h), the transition between the two regimes occurs at a base excitation equal to 0.5*g* confirming the behavior shown by most of the specimens.

### Experimental modal analysis

The FRCs capture very accurately the nonlinear dependence of the storage modulus with the deformation level. As seen in Fig. 2, the backbone curves describe how the nonlinear frequency depends on the oscillation amplitude. Since the modal mass does not change with the oscillation amplitude, the variation of the nonlinear frequency with the amplitude reflects the variation of the storage modulus with the amplitude. At the same time, the amplitude of the harmonic response of the samples at resonance depends on the loss modulus. Thus employing the half power bandwidth method yields a good estimate of the loss modulus. We thought that a different approach to the estimation of the storage and loss modulus could rely on experimental modal analysis (EMA). EMA typically yields the modal frequencies and damping ratios under various excitation signals. However, instead of running the analysis at a fixed driving amplitude, we employed EMA to determine the frequency and damping ratios upon increasing the excitation amplitude so as to drive the material sample through its nonlinear resonances. A periodic chirp signal was applied in the frequency range 100 Hz to 2 kHz. This range covers the frequency bandwidth of the lowest three modes, namely. the first and second bending flapping modes (i.e., deflections in the thickness-wise direction) and the first lagging mode (i.e., deflections in the width direction). Despite the excitation was applied in the flapping wise direction, the geometrical imperfections of the beams were such that the lowest lagging mode got also excited. The tests were performed by driving the shaker in open loop while the excitation amplitude was regulated by the input voltage. An accelerometer was placed on the shaker head expander to acquire the excitation signal and evaluate the FRFs via the EMA approach. Several acquisitions were performed increasing the input voltage from 0.01 to 0.3 V with the purpose of obtaining the evolution of the resonance frequency and damping ratio with the excitation amplitude. For the type of considered excitation and the open loop control, it was not possible to establish a correlation between the applied voltage and the base acceleration amplitude as done for the frequency sweep tests. However, this procedure represents an innovative experimental approach that may be suitably applied to describe the nonlinear trend of the resonance frequencies and damping ratios in other experimental contexts.

The frequencies and damping ratios of the lowest three modes are reported in Table 3 for $\text{ S}_{2}$ at various excitation levels. The investigated frequencies exhibit a softening behavior while the increase of the damping ratio with the excitation is clearly observed only for the first and second modes. The damping associated to the second mode is almost constant while for the third mode it decreases with the excitation amplitude. The measured resonance frequency of the first mode is close to the values acquired via the frequency sweep tests.

**Table 3:** Frequencies and damping ratios of the lowest three modes obtained under a periodic chirp with increasing amplitude for sample $\text{ S}_{2}$ containing 0.25 wt% bCNT.

Voltage (V)	First mode		Second mode		Third mode	
	$\ f_{n}$ (Hz)$^{\rm a}$	$\zeta$ ($\%$)$^{\rm b}$	$\ f_{n}$ (Hz)$^{\rm a}$	$\zeta$ ($\%$)$^{\rm b}$	$\ f_{n}$ (Hz)$^{\rm a}$	$\zeta$ ($\%$)$^{\rm b}$
0.01	214.5	0.525	1314.8	0.552	1925.4	0.221
0.05	214.2	0.592	1310.3	0.554	1924	0.166
0.1	213.5	0.617	1307.3	0.540	1924.3	0.109
0.2	212.9	0.660	1299.3	0.526	1922.8	0.101
0.3	212.5	0.725	1293.7	0.465	1923.2	0.086

^a^Resonance frequency.

^b^Damping ratio.

## Discussion

The experimental campaign was aimed to investigate the nonlinear dynamic properties in terms of frequency response and damping ratios of PBT/bCNT nanocomposite cantilevers. Eight samples with different bCNT content were tested to acquire the FRCs of the lowest modes while the damping ratios were estimated using the half power bandwidth method. In addition, for cross-validation purposes, EMA analysis was applied in an unprecedented way to identify the resonance frequencies and the damping ratios of the lowest three modes upon increasing the excitation amplitude. The results confirm some of our previous experimental observations and theoretical predictions which highlight the key role of the interactions of the single- or multi-walled CNTs with the thermoplastic polymer chains surrounding them.

However, the present tests with the PBT/bCNT samples revealed surprising new results associated with this type of branched MW-CNTs. To sort out the different sources of nonlinearities, usually the specimen are tested under suitable boundary conditions and excitation amplitudes that will induce geometric nonlinearities only (e.g., stretching nonlinearity, curvature nonlinearity, etc.) in contrast with material nonlinearities. In the present testing conditions, the geometric hardening of the lowest mode of the cantilevered samples is due to the nonlinear bending curvature while the nonlinearity due to the stick-slip mechanism between CNTs and polymer chains induces a softening of the response. The softening trend observed for all samples with bCNT wt% ranging from $0.1$ $\%$ to $1$ $\%$ suggests that the stick-slip mechanism is robust enough to overcome the geometric curvature-induced hardening. Moreover, the trend of the damping ratio and the transition of the FRCs from single- to multi-valued responses and viceversa highlight different regimes characterized by the occurrence or by the non activation of the interfacial stick-slip. On the other hand, the softening response at low amplitudes followed by a hardening response at higher amplitudes, observed for the two samples with 2 wt% bCNT, can be explained by the fact that the material softening at low oscillation amplitudes when the stick-slip is activated is overcome by the curvature-induced geometric hardening at higher oscillation amplitudes which is emphasized by an additional, powerful hardening effect induced by the stretching of the bCNTs network which behaves as a membrane embedded in the polymer matrix. However, the trend of the damping ratio, the transition towards single-valued FRCs and the strong hardening highlight the fact that the material nonlinearities play a role in modifying the original hardening exhibited by the polymeric samples without the integration of CNTs. The results obtained via EMA suggest the occurrence of rearrangements of the bCNTs within the hosting matrix due to the sweep tests. Nevertheless, these aspects require further deep investigations also accounting for the detection of the temperature changes of the samples.

The remarkable behavior exhibited by bCNT/PBT specimens makes this material an excellent candidate for applications requiring high mechanical/damping performance and multifunctional features^11^. The ability to tailor the softening and hardening response by adjusting the bCNT weight fraction can be leveraged to manufacture high-performance materials for the next generation of slender structures capable of withstanding large nonlinear deformations while dissipating significant amounts of energy without suffering damage or failure. In the realm of vibration control, the potential to manipulate the mechanical properties of the composite by varying the bCNT weight fraction can be utilized to create nonlinear metamaterials with secondary bandgaps arising from parametric and sub/super-harmonic resonance phenomena^30^. Additionally, the unusual switch from softening to hardening can be harnessed for the development of new generations of micro-sensors. Overall, the unique and highly tunable properties of bCNT/PBT composites make them a promising material for a wide range of engineering applications, particularly those requiring advanced mechanical performance and multifunctionality.

## Methods

### Samples preparation and morphological characterization

**Figure 3:**
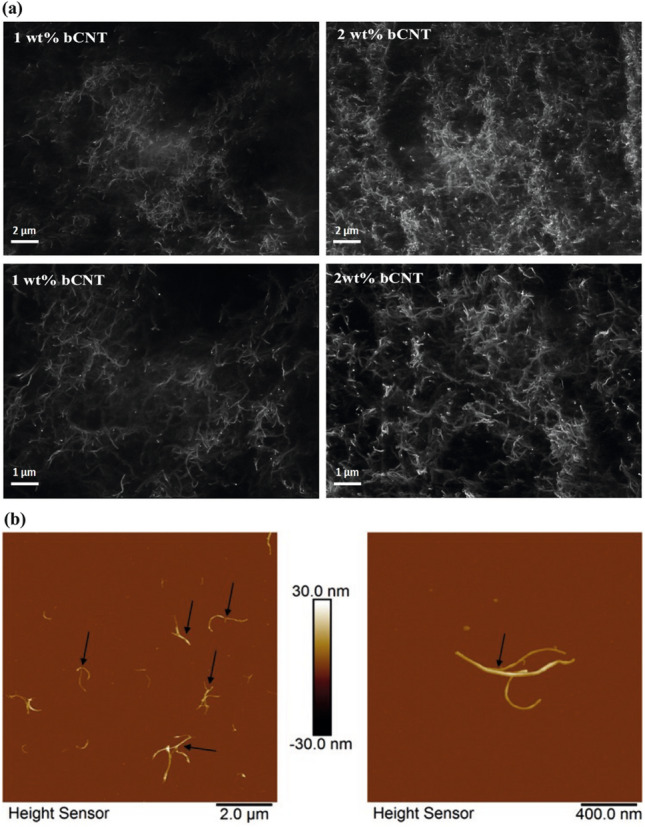
(**a**) The SEM-CCI image of the melt-mixed PBT/1 wt% and 2 wt% b-MWCNT nanocomposites shows a homogeneous distribution of b-MWCNT (visible as light gray lines while the polymer matrix is black) without large residual agglomerates. (**b**) AFM image of dissolved b-MWCNTs from the PBT matrix (1$\%$ wt composite), arrows indicate the points of branching of the b-MWCNTs.

The polymer used in the preparation of the nanocomposite samples was PBT Vestodur 3000 (Evonik Industries, Marl, Germany) with a melt flow rate of 9 cm^3^/10 min (250 $^{\circ }$C, 2.16 kg) while the filler was b-MWCNT CNS-PEG (Applied NanoStructured Solutions LLC, Baltimore, MD, USA)^31^. The composites were produced by direct incorporation of bCNTs (0.25 wt%, 0.5 wt%, 1 wt%, 2 wt%, see Fig. 3) by means of melt mixing in a small-scale conical twin-screw micro compounder Xplore 15 (Xplore Instruments BV, Sittard, The Netherlands) having a volume of 15 cm. By following the fabrication steps reported in^32^, a temperature of 265 $^{\circ }$C, a rotation speed of 200 rpm and a mixing time of 5 min were selected as processing conditions. The composites were compressed and molded to sheets (45 mm $\times$ 10 mm $\times$ thickness 1 mm) using the hot press PW40EH (Paul-Otto Weber GmbH, Remshalden, Germany) at 265 $^{\circ }$C for 1 min.

The morphological characterization was performed using scanning electron microscopy (SEM) Carl Zeiss Ultra plus. The visualization of the bCNT network was performed on compression molded plates using SEM in charge contrast imaging mode (InLens detector at 20 kV). The characterization of the single CNTs was performed by means of atomic force microscopy (AFM) dissolving in trifluoroacetic acid (CAS 76-05-1) 1 wt% bCNT/PBT for 1 hour at room temperature. A drop of dispersion was set on freshly cleaned mica surface and allowed to evaporate. The AFM measurement was performed like that described in Talò et al.^7^. The measurements were acquired in peak force tapping mode thanks to a Dimension FastScan (Bruker-Nano, USA). A silicon nitride sensor ScanAsyst-FLUID+ (Bruker, USA) with a nominal spring constant of 0.7 N/m and tip radius of 2 nm was used. The macrodispersion of CNTs in the hosting matrix was investigated by a transmission light microscopy on sections 5 $\upmu \text{ m }$ thick obtained by strands extruded at room temperature with a microtome RM2265 (Leica Mikrosysteme Vetrieb GmbK, Bensheim, Germany) equipped with a diamond knife. The cuts were fixed on glass slides using the aqueous mounting medium Aquatex$\circledR$ (Sigma-Aldrich, Steinheim, Germany).

The LM study was performed with a microscope BX53M combined with a camera DP74 (Olympus Deutschland GmbH, Hamburg, Germany). For the preparation of the high mechanical performance polymer, a homogeneous dispersion of the bCNT in polymeric matrix is required. This is achieved by the shear stress during melt compounding^33,34^ which yields very good, uniform dispersion. The formation of a network (all our PBT/CNT nanocomposites were found to be electrically conductive thus proving that a network of CNTs was indeed formed) of interconnected CNTs that transfers the mechanical stress and the electrical current explains the differences in electrical, rheological, and mechanical properties in polymer nanocomposites compared to the pure baseline polymers^35,36^. The mechanical forces are transmitted equivalently through the CNT network, the polymer matrix and the CNT-polymer network. In contrast, the electrical current is transferred exclusively in the CNT network and its connection contacts. Due to the high specific surface area of the CNTs, a large interphase is formed between the CNTs and the polymer matrix, which can contribute to the improvement of the mechanical properties such as the impact strength, notched impact strength or, in some cases, the stiffness and elongation at break^33^. The electrically conductive network and thus the CNTs are made visible in SEM-CCI images Fig. 3a as a light grey area while the polymer matrix is black. As expected, it can be seen in the images that the network is clearly denser with a higher CNT content. Particularly in the PBT composite with 1 wt% b-MWCNT, black regions are also visible between the cloud-like CNT-containing areas, indicating the typical secondary agglomeration of CNTs in the nanoscale range^34^. Due to the higher filling level in the PBT/b-MWCNT 2 wt% composite, this structure is barely visible.

For the electrical measurements, the PBT/b-MWCNTs composites are electrically conductive at the concentrations investigated, namely, 0.25 wt% 1 Ohm cm, (with 0.5 wt%, 2.3E Ohm cm, with 1 wt%, 633 Ohm cm, with 2 wt%, 470 Ohm cm). This means that in all composites there is a network of CNTs that can significantly influence the mechanical properties of the composite. An increase of melt viscosity followed by hardening with increasing CNT content has been observed for polymer/CNT composites^37–39^.

### Experimental campaign

**Figure 4:**
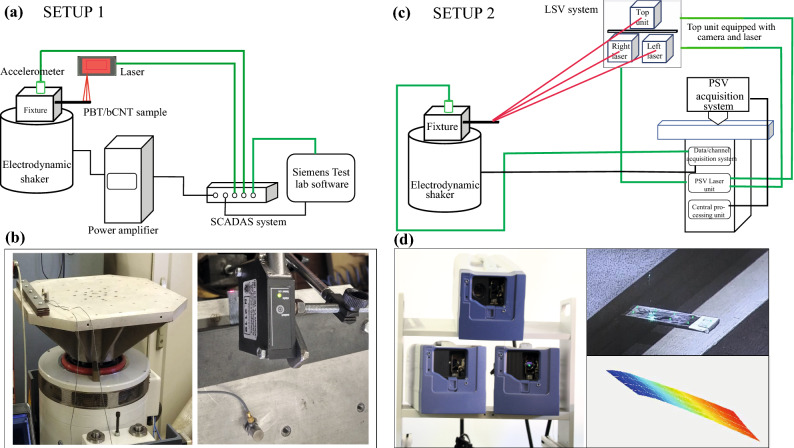
Schematic view of the two experimental setups: (**a,b**) show the setup to acquire the FRCs at the tip of the tested nanocomposite samples subject to an electrodynamic shaker with the single-point laser displacement transducer; (**c,d**) show the setup for the EMA analysis using the 3D Polytec scanning vibrometer system (view of the three laser heads), a view of the sample and an example of a modal output (the first bending mode).

The nanocomposite beams employed for the experimental campaign are constituted by bCNTs with wt% equal to 0.25%, 0.5%, 1%, and 2%. For each wt%, two specimens were considered obtaining a total number of eight samples as shown in Fig. 1. The beams are 44 mm long with a rectangular cross section of width equal to 9.8 mm and a thickness of 1 mm. Each sample was arranged in cantilever configuration obtaining a span equal to 32.5 mm. Particular attention was paid to the effectiveness of the clamp. The sample was mounted on an electrodynamic shaker that applies the base excitation in the thickness direction as shown in Fig. 4.

### Frequency sweep tests

The employed shaker with operational range up to 10 g and 2 kHz shown in Fig. 4a,b is controlled by the Siemens Testlab software with a feedback accelerometer (PCB electronics with full scale of 50 g). A laser displacement sensor (optoNCDT 1320 produced by Micro-Epsilon) measures the oscillation amplitude of the sample tip. It can measure the deflections within the range of ± 10 mm and a resolution of 0.02%. The described setup applies a base acceleration to the beam and acquires the absolute displacement of the tip. The acquired displacement quantity can be assumed to be a fair estimate of the relative displacement considering the frequency and acceleration ranges for which the experiments were performed.

The objective of the experimental campaign was to obtain the FRCs of the samples for increasing levels of the base acceleration near the resonance of the lowest natural frequency. To this end, stepped sine sweeps were performed with a frequency resolution equal to 0.1 Hz. In order the ensure a steady-state response, the specimen was excited at the same excitation frequency for a sufficiently large number of cycles comprised between 500 and 1000 cycles. The last 10 cycles were employed to estimate the amplitude and the phase of the response using the harmonic estimator implemented in Siemens Testlab. The harmonic estimator extracts the amplitude and the phase for each excitation frequency by minimizing the difference between the acquired displacement and a sine function. In this way, the frequency and phase spectra as well as the Frequency response functions (FRFs) can be recorded.

For certain levels of excitation, the beams exhibited a jump or multiple jumps in the response caused by fold bifurcations. In these cases, both forward and backward sweeps were performed in order to capture the full set of the stable responses close to resonance. Finally, the half power bandwidth method was employed to estimate the damping ratio associated with each FRC obtaining the trend of the equivalent damping ratio as function of the excitation level.

### Experimental modal analysis

The Polytec (PSV-500-3D) LSV system was used to acquire the operational deformed shapes exhibited by the beams within a frequency range up to 2000 Hz. The velocities of the beam acquisition points belonging to a rectangular grid defined on the top surface were recorded. The operating principle of the PSV is based on the Doppler effect and the use of an interferometer to combine the measurement laser beam with the back-scattered laser beam which is perturbed by the motion. The PSV-500-3D system makes use of three scanning heads (see Fig. 4d). The back-scattered light undergoes a frequency shift proportional to the velocity of the grid moving point and a photo detector records the interference. Finally, a velocity decoder provides the measurement. For these tests, the shaker was controlled in open loop with the signal generator of the PSV. Several chirp signals with increasing amplitude from 0.01 to 0.4 V were applied up to 2 kHz to capture also the high frequency modes. The velocity estimates were performed according to four or five averages on each scanned point. The EMA analysis was conducted with the PolyWave software applying the Complex Mode Indication Function (CMIF) method on the operational deformed shapes of the beams. The CMIF is based on the singular value decomposition of the FRF matrix at each spectral line, and it provides modal parameters, such as damped natural frequencies, mode shapes, modal dampings and modal participation vectors. EMA is well known in the literature for the estimation of the linear modal properties. However, it is here applied for the first time with the purpose of detecting the nonlinear trends of the resonance frequencies and modal damping ratios. For this reason, several acquisitions were performed increasing the amplitude of the chirp signal and identifying the values of the resonance frequencies and damping ratios for the lowest three modes. This approach can be considered as an equivalent linearization of the beam response to different excitation amplitudes.

### Theoretical predictions

The experimentally observed unique behavior discussed in “Results” and shown in Fig. 2 was predicted by a nonlinear beam model incorporating a constitutive hysteretic law for CNT/polymer nanocomposite material presented in^29^. The nonlinear beam model was derived consistently from a 3D mesoscale constitutive model of hysteresis for the CNT/polymer composite^8,9^ through a mechanical reduction process employing the uniaxial strain state ansatz in plane bending which yields a hysteretic moment-curvature relationship. Such moment-curvature relationship (*M*-$\kappa$) can be cast as a direct summation of a linear contribution and a hysteretic contribution $\chi$ mimicking in the well-known Bouc-Wen model:

1 $$\begin{aligned} \begin{aligned} {M}&= (EJ)_1 \, [ \delta \,{\kappa } +\left( 1-\delta \right) \chi ] \\ \dot{\chi }&= \left[ 1 - \left( \bar{\beta }+\bar{\gamma }\,\text{ sign }(\chi \dot{\kappa }) \right) |\chi |^n\right] \dot{\kappa } \end{aligned} \end{aligned}$$

where $\delta := (EJ)_2 \big / (EJ)_1$ is the ratio between the post-slip bending stiffness and the elastic bending stiffness, and $(\bar{\beta },\bar{\gamma })=({\beta },\gamma )/\kappa _y^n$ with $\kappa _y=M^\text {(o)}/[(1-\delta ) \, (EJ)_1]$ denote the yielding bending curvature, $M^\text {(o)}$ the yielding moment, and $(\beta ,\,\gamma ,\,n)$ parameters governing the shape of the hysteresis loops. Note that both the elastic and post-elastic bending stiffness coefficients depend on the Young moduli $E_{(p,c)}$ and Poisson’s ratios $\nu _{(p,c)}$ of the two phases and on the CNT weight fraction (see^29^ for more details) according to the formulas:

2 $$\begin{aligned} \begin{aligned} (EJ)_1&= (EJ)_p + \widehat{EJ}_1\left( E_{(p,c)},\,\nu _{(p,c)},\,\text {CNT wt\%}\right) \\ (EJ)_2&= (EJ)_1 + \widehat{EJ}_2\left( E_{(p,c)},\,\nu _{(p,c)},\,\text {CNT wt\%}\right) \end{aligned},\quad \text {with }\widehat{EJ}_2<\widehat{EJ}_1 \end{aligned}$$

where the subscript *p* stands for polymer and *c* for CNT, respectively.

**Figure 5:**
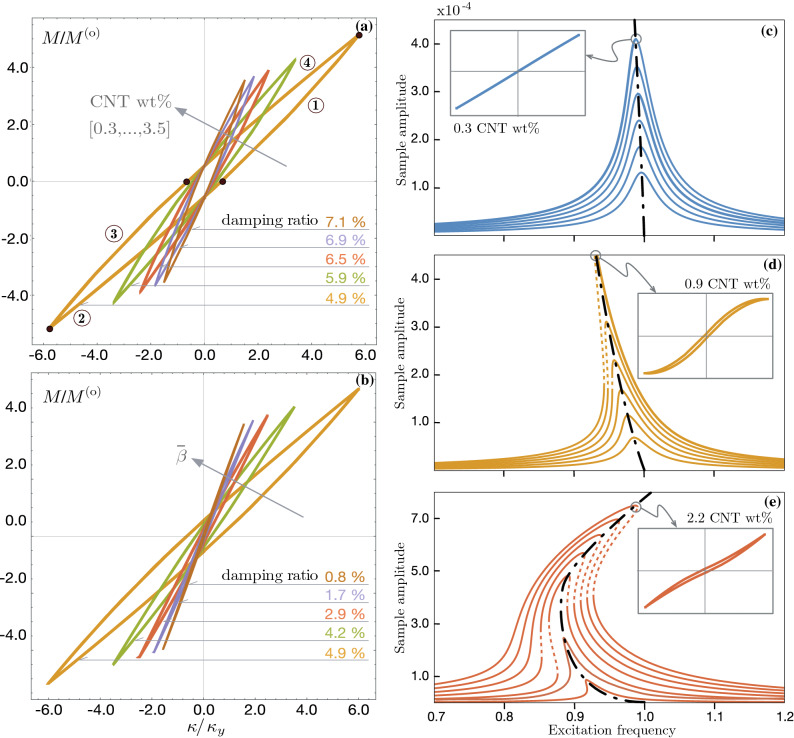
(**a,b**) Families of moment-curvature constitutive responses of the nanocomposite material upon variations of the CNT wt% and the parameter regulating the interfacial shear strength $\bar{\beta }$. Families of frequency response curves of the lowest bending mode predicted by the PBT/bCNT model containing (**c**) 0.3 wt%, (**d**) 0.9 wt%, (**e**) 2.2 wt% bCNTs for increasing excitation amplitudes. The FRCs are computed via the asymptotic approach developed for the hysteretic nonlinear model presented in^29^. The solid (dashed) curves indicate stable (unstable) periodic responses.

We used a novel approach by combining the Galerkin discretization approach with the method of multiple scales to represent the restoring force in each of the four branches of the hysteresis loops. This approach enabled us to develop a piece-wise ordinary differential equation (ODE) to predict the asymptotic periodic solutions and frequency response functions for the primary resonance of the first bending mode (as illustrated in Fig. 5).

We then plotted the constitutive responses and FRCs for different values of CNTs wt% and increasing excitation amplitudes (Fig. 5a,b). Our results showed that the curves exhibited a predominantly softening nonlinearity that transitioned to a hardening characteristic response for higher CNT wt% and at higher oscillation amplitudes (Fig. 5c–e).

Our model revealed the competing effects between two antagonistic factors: the material softening induced by the CNT/polymer interfacial stick-slip, and the geometric hardening due to the nonlinear bending curvature of the cantilever beam. We found that the geometric hardening can become dominant over the material softening when large oscillation amplitudes are reached. Moreover, the formation of a network of bCNTs at higher wt% acted as a reinforcing network inside the beam sample, contributing to the hardening mechanism.

Overall, our analytical predictions and experimental results demonstrated excellent qualitative agreement, emphasizing the highly tunable and nonlinear mechanical response of this class of nanostructured materials. This behavior suggests that these materials can be strategically tailored depending on the applications and range of operation, making them suitable for high-performance applications such as slender structures capable of sustaining large nonlinear deformations, micro sensors, and nonlinear metamaterials with secondary bandgaps.

## Data Availability

The data that support the findings of this study are available from the corresponding author upon reasonable request (walter.lacarbonara@uniroma1.it).
